# A Machine Learning-Guided Study of Structure–Reactivity Relationships in Diels–Alder Cycloadditions

**DOI:** 10.1021/acs.joc.5c02349

**Published:** 2026-01-07

**Authors:** Amir Mahdian, Kaveh Farshadfar, Kari Laasonen

**Affiliations:** Department of Chemistry and Material Science, School of Chemical Engineering, 174277 Aalto University, Espoo 02150, Finland

## Abstract

The Diels–Alder cycloaddition is a cornerstone transformation in organic synthesis and has been extensively studied in both experimental and theoretical contexts. In this work, we present a complementary computational approach that combines density functional theory (DFT) and machine learning to further elucidate the role of steric and electronic effects in determining the reactivity and activation barriers. A diverse dataset of 1000 uncatalyzed hydrocarbon Diels–Alder reactions was used to train predictive models that relate activation energies to chemically meaningful molecular descriptors. SHAP analysis of the machine learning models highlights the dominant influence of steric effects, particularly those associated with substituent volume at the internal diene carbons, which can impose conformational strain and lead to significantly elevated barriers. In contrast, substituents at the terminal positions have a more limited impact. We introduced the minimum energy gap between LUMO_diene_–HOMO_dienophile_ and LUMO_dienophile_–HOMO_diene_ as a key predictive descriptor. This feature shows a strong correlation with the activation energy across the dataset, although steric interactions can lead to notable deviations from the overall trend. The resulting models provide insights for rationalizing selectivity and designing more efficient cycloadditions based on steric and electronic complementarity.

## Graphical abstract

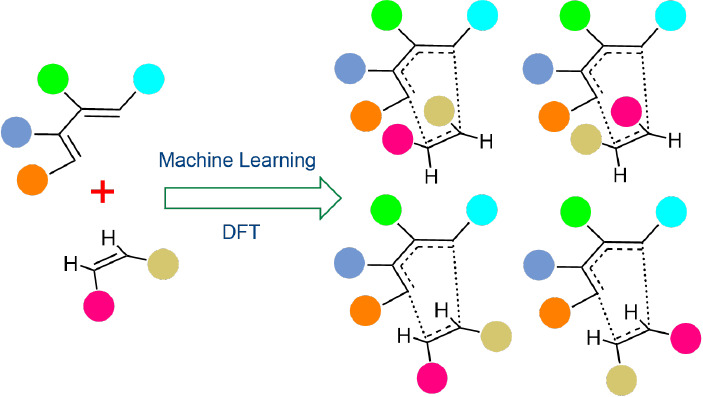

## Introduction

The Diels–Alder cycloaddition serves as a fundamental tool in organic synthesis, facilitating rapid assembly of six-membered ring systems commonly encountered in complex frameworks.^1^ This pericyclic transformation involves the formation of two σ-bonds concomitant with the cleavage of two π-bonds, affording structurally diverse products with well-defined stereochemical and regioselective outcomes.^2–4^ Over the years, the mechanistic intricacies of this transformation have been extensively studied. Key areas of focus include whether the reaction proceeds through a concerted or stepwise mechanism, the synchronicity of the transition state, and the effects of substituents on both the diene and the dienophile in affecting the activation energy and determining regio- and stereoselectivity.^5–9^

In this context, Houk and coworkers established two general principles for predicting regioselectivity based on the magnitudes of frontier orbital coefficients and the relative energies of the interacting orbitals at the transition state.^10^ Building upon this foundation, Hirao and collaborators introduced the Reactive Hybrid Orbital (RHO) approach, which offers improved accuracy over conventional frontier orbital theory by explicitly accounting for orbital mixing in the transition state.^11–13^ While frontier molecular orbital theory is widely accepted as the dominant feature governing regioselectivity, steric effects, often considered secondary, can be significant, as ortho products are frequently favored over less hindered meta isomers.^14–16^

Although recent studies have applied machine learning (ML) approaches to a variety of Diels–Alder reactions, including inter- and intramolecular processes^17–20^ transformations involving heteroatoms such as nitrogen^21^ and catalyzed reactions^19,22^ to the best of our knowledge, only a few studies^23^ have explored predictive ML models specifically tailored for uncatalyzed Diels–Alder reactions between hydrocarbons.

This study aims to develop unbiased predictive machine learning (ML) models for uncatalyzed Diels–Alder (DA) reactions. We assess the performance of ML approaches combined with density functional theory (DFT) calculations in predicting key reaction parameters, as guided by the SHAP value interpretation. The work centers on DA cycloadditions involving structurally diverse substituents, which constitute a representative dataset for training models to estimate activation barriers from computed chemical descriptors.

## Results and Discussion

As illustrated in Scheme [Fig usch1], bond formation can occur between C^1^ and C^5^, between C^4^ and C^6^, or with reversed diene-dienophile alignment, resulting in two distinct regioisomeric pathways. Each of these can proceed via either an endo or exo transition structure, ultimately leading to four distinct reaction pathways and four possible cycloadducts. To probe substituent effects, nine representative groups, including OMe, ^*t*^Bu, Me, phenyl, H, F, COOMe, CHO, and CN, were introduced at C^1^, C^4^ (diene), and C^5^, C^6^ (dienophile), which directly engage in the bond-forming step of the DA reaction. These substituents span a broad range of electronic and steric properties, including electron-withdrawing groups (EWGs), electron-donating groups (EDGs), and bulky substituents, such as ^*t*^Bu. A subset of substituents comprising F, CN, OMe, ^*t*^Bu, and H was introduced at the C^2^ and C^3^ positions of the diene, which were initially assumed to exert a limited influence on the overall reactivity.

**1 usch1:**
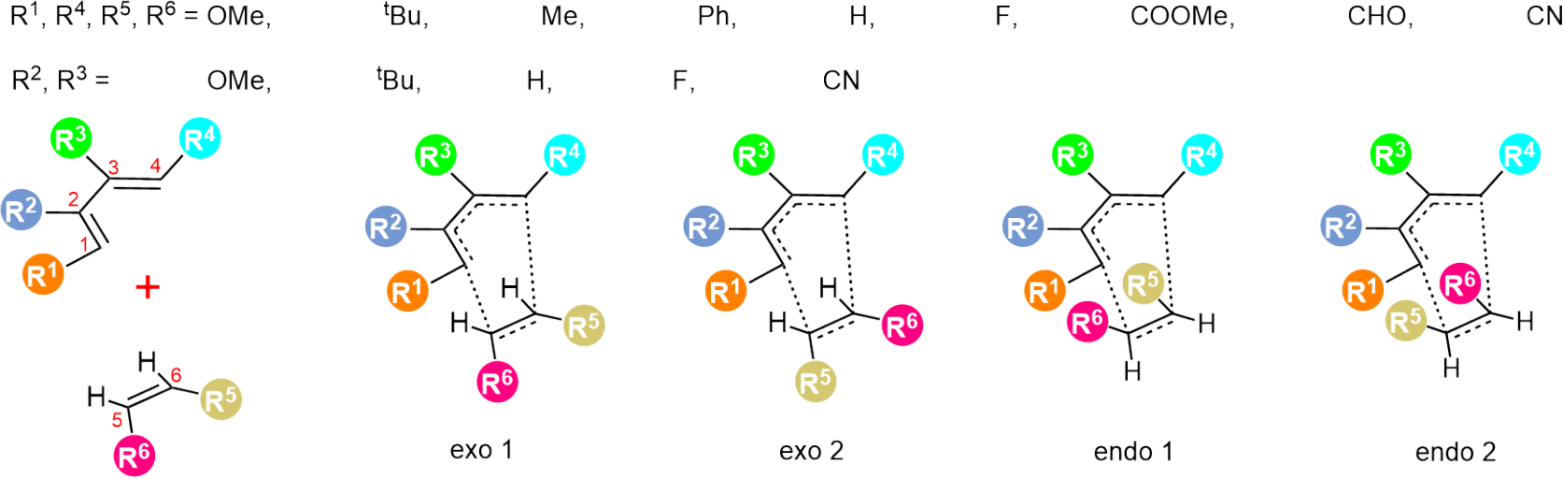
Endo and Exo Pathway Analysis in Diels–Alder Reactions: Four Stereochemical Potential Products by Substituents

Using these substituents, a total of 91,044 distinct Diels–Alder reactions (each reaction may proceed through up to four distinct pathways, although in cases where symmetry is present, fewer unique pathways exist) were generated. From this dataset, a random subset of 1000 unique reactions was selected to ensure balanced representation across all substituent classes. All transition structures and their corresponding diene and dienophile substrates were optimized at the M06-2X/6-31G­(d) level of theory. Single-point energy refinements were subsequently performed by using the def2-TZVP basis set.

### Machine Learning Approach

Among all possible transition structures illustrated in Scheme [Fig usch1], the one with the lowest activation free energy $\left(\Delta G_{\min}^{‡}\right)$ was selected as the target variable. To assess how electronic and steric properties of substituents on the diene and dienophile influence the activation barrier, a set of molecular descriptors was evaluated to model the effect of substituents on the activation barrier. Accordingly, we examined correlations across 37 descriptors capturing both steric and electronic features to assess their predictive relevance to the activation barrier.

The selected features include the frontier orbital energies—highest occupied molecular orbital (HOMO) and lowest unoccupied molecular orbital (LUMO)—of both the diene and dienophile; frontier energy gaps (LUMO_diene_–HOMO_dienophile_ and vice versa); and min_LUMO–HOMO_, defined as the minimum of the two frontier gaps. The population of the p_*z*_ orbital, which is oriented perpendicular to the molecular plane and participates in the cycloaddition process, was also included as a descriptor due to its potential influence on reactivity. Additional features comprise the Hammett σ_p_ parameter (quantifying electron-donating or -withdrawing effects), natural population analysis (NPA) charges at key atoms within the DA backbone, and steric descriptors such as substituent volumes and sterimol parameters—multidimensional metrics that capture conformationally averaged size and shape.^24^ Detailed definitions of all descriptors are provided in the Supporting Information (Table S1).

These descriptors were used as input features for training both random forest (RF) and gradient boosting (GB) regression models.^25^ Details of alternative ML approaches are provided in (Supporting Information S2). Initial models were trained using the full feature set with both random forest and gradient boosting regressors. SHAP analysis revealed that several descriptors exhibited weak correlations with the target variable and reduced predictive performance. These features were removed, and the models were retrained using 13 high-importance descriptors, including the substituent volume, min_LUMO–HOMO_, and σ_*p*_ parameters.

After 50 iterations (evaluated as the average over 50 train/test splits), the gradient boosting regression model exhibited an inferior predictive performance, as shown in Figure 1. The model achieved *R* ^2^ values of 0.95 and 0.88 for the training and validation sets, respectively, with a mean absolute error (MAE_test_) of 2.24 kcal/mol. Although the gradient boosting regression model achieved reasonably high accuracy, the random forest model outperformed it, delivering a lower prediction error and improved consistency across training, validation, and out-of-bag evaluations. Therefore, the RF was selected for further analysis due to its greater robustness and predictive reliability.

**Figure 1:**
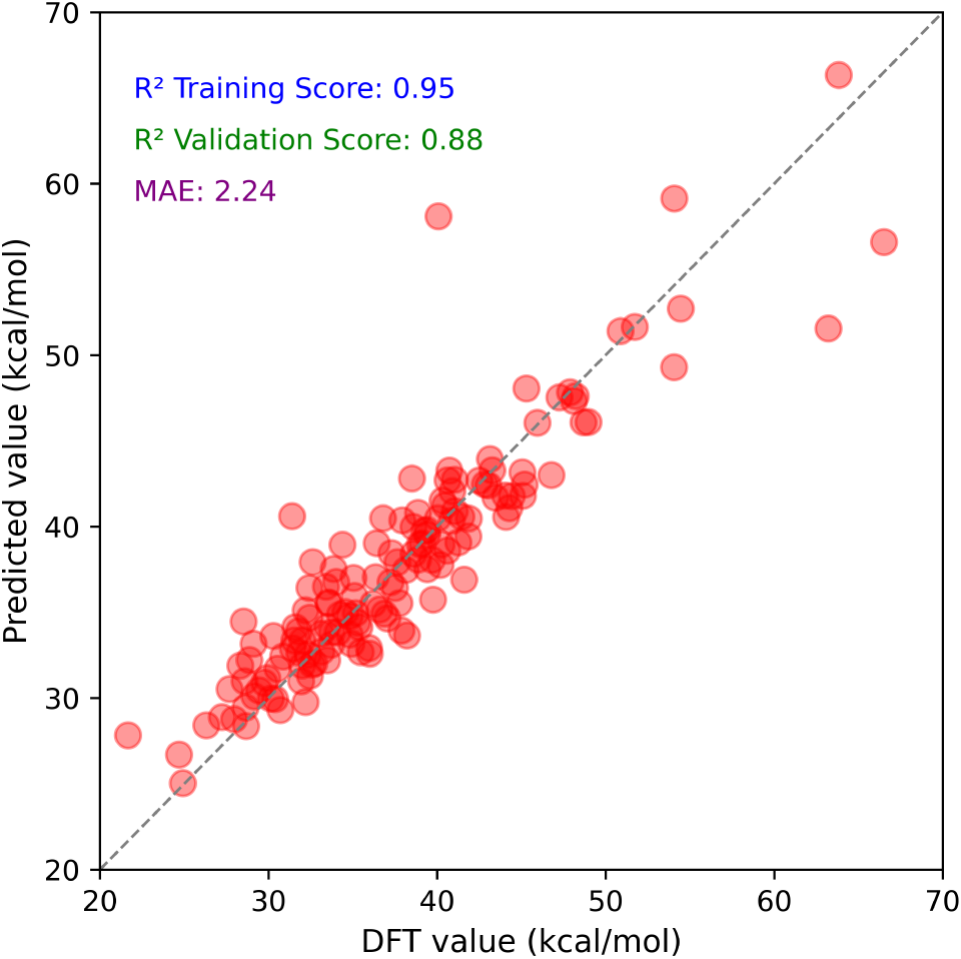
Scatter plots of DFT-computed $\Delta G_{\min}^{‡}$ versus predicted values from the gradient boosting (GB) regression model, trained on 13 attribute-based features.

Figure 2 illustrates the predictive performance of the optimized random forest regression model, which comprised 600 decision trees. Node splitting was allowed with a minimum of two samples, and terminal nodes were permitted to contain at least one sample. Model performance was evaluated by using out-of-bag (OOB) error estimation to avoid dependence on a separate test set. Hyperparameters were optimized via 10-fold cross-validation to improve the generalizability and reduce overfitting. A full description of the hyperparameter optimization procedure, together with the corresponding optimized parameter values, is provided in the Supporting Information (Tables S2 and S3). The final model achieved *R* ^2^ scores of 0.96 training, 0.96 validation, and 0.84 OOB, along with a mean absolute error (MAE) of 1.30 kcal/mol. These metrics indicate that the selected input features effectively capture the relevant patterns and underlying reactivity trends.

**Figure 2:**
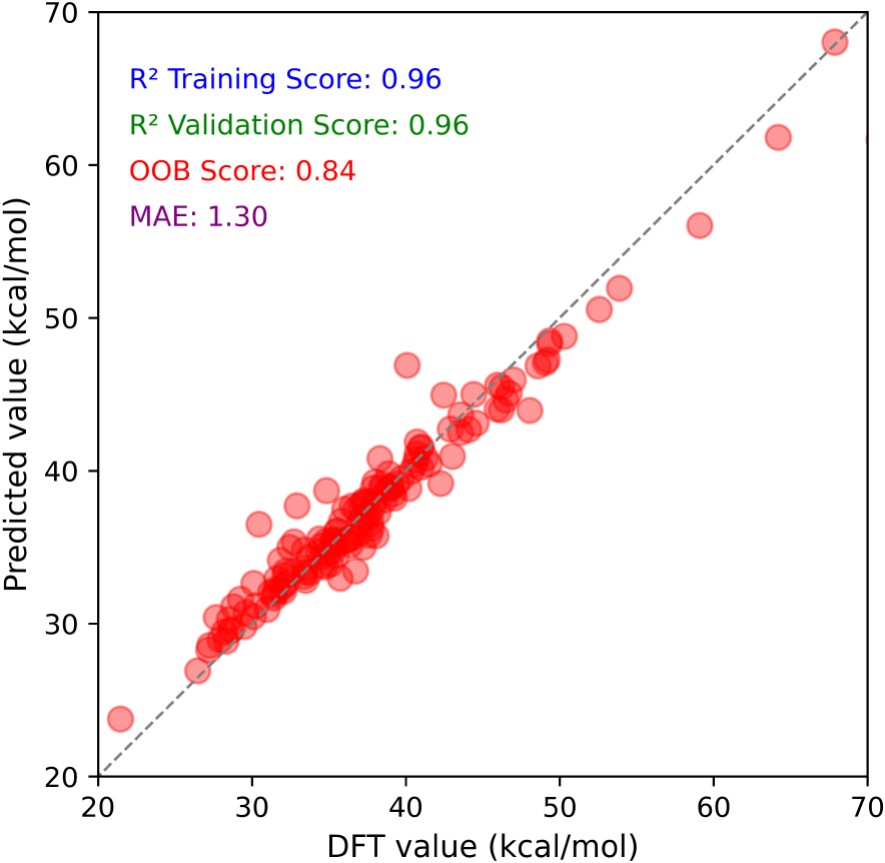
Scatter plots of DFT-computed $\Delta G_{\min}^{‡}$ versus predicted values from the random forest (RF) regression model, trained on 13 attribute-based features.

### Most Important Parameters

SHapley Additive exPlanations (SHAP) analysis^26^ was employed to assess feature importance and interpret the machine learning model. This analysis is based on the principle of breaking down a model’s prediction by attributing contributions to each feature, accounting for its absence. This breakdown reveals the importance of each feature in determining the model’s final prediction of activation energy (SHAP values represent the deviation of each sample’s predicted $\Delta G_{\min}^{‡}$ from the average predicted $\Delta G_{\min}^{‡}$ across all samples). Further details of the descriptor correlation analysis, including the complete Pearson correlation matrix for all DFT-based features, are provided in the Supporting Information (Table S4). Additional SHAP analyses performed separately on the training and independent test subsets, confirming the stability of the top-ranked descriptors, are provided in the Supporting Information (Figures S11 and S12).

Frontier molecular orbitals, particularly the HOMO and LUMO, have long been documented as central determinants of Diels–Alder reactivity.^10–13,27,28^ As illustrated in Figure 3, the HOMO–LUMO gap emerged as one of the most influential features in predicting the activation free energy $\left(\Delta G_{\min}^{‡}\right)$ . Building on prior studies, Diels–Alder reactions are typically categorized into two principal types: (i) normal DA reactions, wherein the diene bears an EDG and the dienophile features an EWG, resulting in LUMO_dienophile_–HOMO_diene_ dominated interactions; and (ii) inverse DA reactions, in which the diene carries an EWG and the dienophile contains an EDG, with the reaction governed by LUMO_diene_–HOMO_dienophile_ interactions.^29–35^

**Figure 3:**
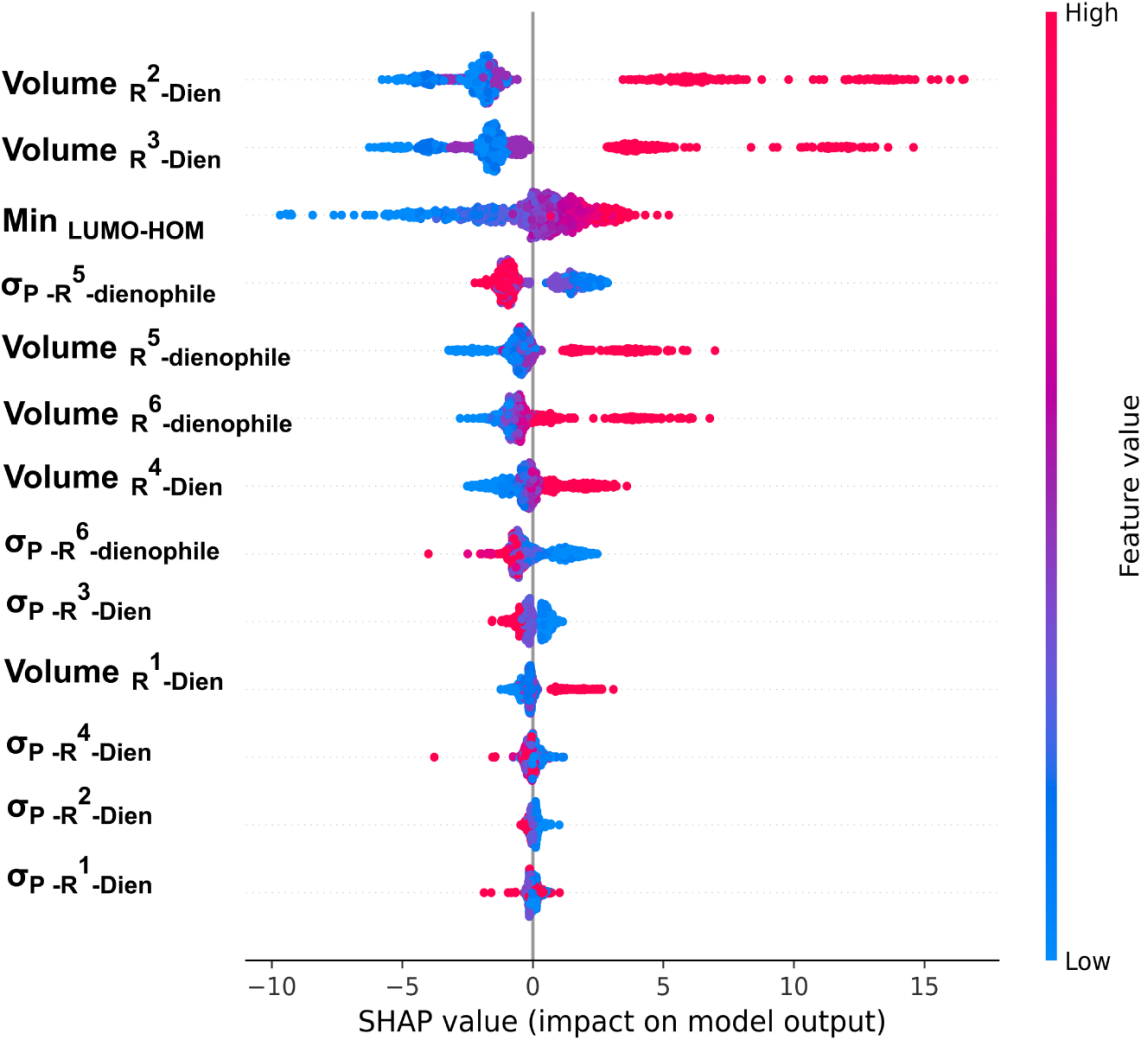
SHAP values of the barrier (kcal/mol) representing the contribution of different features across various samples. Color gradients reflect the feature values, with blue indicating low and red indicating high values.

Analysis of the LUMO–HOMO energy gap revealed a near-even split: approximately half of the molecules exhibited gap values above the dataset average, while the remainder fell below. This distribution aligned with the mechanistic classification into normal and inverse DA reactions. Accordingly, the dataset was partitioned, and separate machine learning models were trained for each reaction type based on the dominant frontier orbital interaction. This approach is expected to enable a more accurate prediction of activation energies and to identify key contributing descriptors. For the normal DA reactions, the RF model achieved *R* ^2^ values of 0.98, 0.83, and 0.84 for the training, validation, and out-of-bag (OOB) datasets, respectively. In comparison, the model trained on inverse DA reactions yielded *R* ^2^ values of 0.97, 0.88, and 0.77 for the training, validation, and OOB sets, respectively. Scatter plots and SHAP-based feature attribution analyses for both models are provided in (Supporting Information SI). A narrower HOMO–LUMO gap enhances frontier orbital interactions and facilitates electron flow from the HOMO to the LUMO, thereby lowering the activation barrier. Conversely, a larger gap is associated with weaker orbital overlap and higher activation energy. Notably, combinations of EDG on the diene and EWG on the dienophile, or vice versa, serve to further reduce the HOMO–LUMO gap, enhancing orbital overlap and promoting reactivity. These observations confirm that the machine learning model effectively captures established quantum chemical principles, reinforcing its mechanistic reliability.

A positive SHAP value for σ_*p*_ indicates that EDGs on the dienophile (C^5^ and C^6^) are associated with a slight increase in activation free energy, whereas EWGs contribute to a reduction in the barrier, thereby facilitating the reaction. While this trend aligns with general electronic principles, the effect of σ_*p*_ is affected by steric and conformational factors, making its interpretation more context-dependent. Remarkably, and contrary to prevailing assumptions, Figure 3 reveals that the substituent volumes at C^2^ and C^3^ are among the most influential features affecting $\Delta G_{\min}^{‡}$ . In this study, ^*t*^Bu, as a representative bulky group, significantly elevates the activation barrier, whereas smaller substituents reduce it (ranging from about 17 to −6 kcal/mol in their effect on the activation barrier), underscoring the critical role of steric effects at these diene positions. Substituents at C^5^ and C^6^ (the dienophile positions) have a comparatively modest effect, with bulky groups raising the barrier by approximately 7 kcal/mol and smaller substituents lowering it by about −3 kcal/mol. In contrast, the substituent volume at C^1^ and C^4^ (the terminal diene positions) exerts a lesser influence on the activation barrier, with effects ranging from approximately 4 to −6 kcal/mol. The substantial increase in activation energy caused by ^*t*^Bu substitution at C^2^ and C^3^ prompted a comprehensive investigation of positional effects, as detailed in the following section.

### Uncovering Why Steric Effects at C^2^ and C^3^ Are Key Contributors to Activation Barrier Height

To further probe the influence of bulky groups at the internal diene positions (Figure 4), a ^*t*^Bu group was placed at C^3^ as a representative bulky substituent, while five substituents, including CN, H, F, OMe, and ^*t*^Bu were introduced at C^2^. In order to focus solely on the effects of substitution at positions 2 and 3, all remaining positions were substituted with H. DFT results indicate that bulky substitution at C^2^ has a greater impact on the activation barrier than electronic effects. Excluding ^*t*^Bu, activation energies for the remaining substituents varied modestly (4.5 kcal/mol), likely due to the relatively similar volumes of H (60.96 Å^3^, sterimol = 1.00), F (98.43 Å^3^, sterimol = 1.08), CN (164.18 Å^3^, sterimol = 1.02), and OMe (334.49 Å^3^, sterimol = 1.53). In contrast, the ^*t*^Bu group has a substantially larger volume (636.38 Å^3^, sterimol = 2.25), leading to significantly greater steric hindrance. Substitution with a single ^*t*^Bu group leads to a pronounced increase in activation energy, reaching up to 16.6 kcal/mol relative to the corresponding system substituent H at the same position, highlighting its exceptional steric impact compared to other substituents.

**Figure 4:**
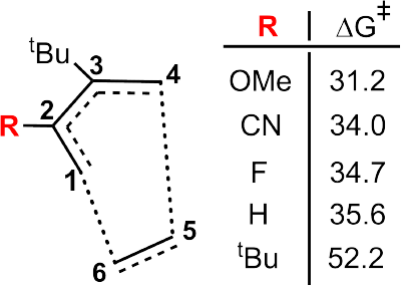
Effect of varying the substituent at C^2^ on the transition structure, with *R* ^3^ substituted with ^*t*^Bu and *R* ^1^, *R* ^4^, *R* ^5^, and *R* ^6^ set to H. All Δ*G* ^‡^ values are given in kcal/mol.

In the second stage of analysis, a ^*t*^Bu group was introduced at C^1^, while the same set of substituents used previously was applied to C^2^, and the resulting activation energies were evaluated. C^1^ can adopt two distinct spatial orientations relative to C^2^: cis and trans. To elucidate the influence of the substituent orientation, both isomers were examined, as illustrated in Figure 5.

**Figure 5:**
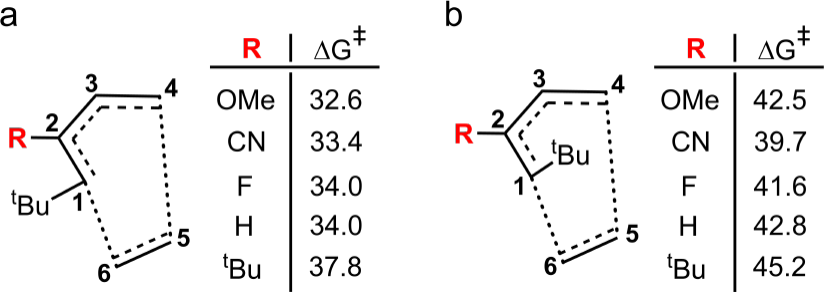
Effect of varying the substituent at C^2^ on the transition structure, with a ^*t*^Bu group substituted at C^1^ (a: cis isomer; b: trans isomer). *R* ^3^, *R* ^4^, *R* ^5^, and *R* ^6^ are substituted with H. All Δ*G* ^‡^ values are given in kcal/mol.

### Effect of Substituent Orientation at C^1^ on Reactivity

The results shown in Figure 5 indicate that when a ^*t*^Bu group is placed at C^1^ of the diene, the presence of the same group at C^2^ has little impact on the activation barrier compared to that when other substituents occupy the C^2^ position. Based on our calculations, the activation barriers for the cis-configured samples range from 32.6 to 37.8 kcal/mol (Figure 5a). In contrast, when the ^*t*^Bu group is positioned on one of the internal carbon atoms of the diene, its steric effect becomes significantly more pronounced, such that placing ^*t*^Bu groups at both C^2^ and C^3^ raises the activation barrier to as high as 52.2 kcal/mol (Figure 4).

In cases in which the ^*t*^Bu group and R are positioned trans to each other (Figure 5b), the overall activation barriers are generally higher than those observed for the corresponding cis isomers. This increase can be attributed to greater steric congestion involving both intramolecular and intermolecular interactions. However, when two ^*t*^Bu substituents are located at positions 1 and 2, the activation barrier is not significantly higher compared to cases where R at C^2^ is a different group, in contrast to the situation where the substituents occupy positions 2 and 3.

The pronounced increase in the activation barrier observed when bulky groups simultaneously occupy the C^2^ and C^3^ positions of the diene, compared to substitution at C^1^ and C^2^, can be rationalized by conformational analysis (Scheme [Fig usch2]). In the ground-state geometry, bulky substituents at C^2^ and C^3^ $\left(G_{0}^{i}\right)$ preferentially adopt a conformation that maximizes their spatial separation, thereby minimizing steric repulsion. However, to participate in the Diels–Alder cycloaddition, the diene must adopt the $\left(G_{1}^{i}\right)$ conformation, which forces both bulky groups to align on the same face of the molecule. This conformational shift results in a significantly higher energy relative to that of the ground state, contributing to the elevated activation barrier. In contrast, when bulky groups are positioned at C^1^ and C^2^ (or symmetrically at C^3^ and C^4^) in a cis configuration, the steric repulsion between them remains similar in both the ground state $\left(G_{0}^{ii}\right)$ and the reactive conformation required for cycloaddition $\left(G_{1}^{ii}\right)$ . As a result, the activation barrier is not substantially increased. For the trans configuration of C^1^ and C^2^ (or C^3^ and C^4^), although the steric repulsion between the two bulky groups remains relatively constant during conformational changes, the substituent at C^1^ introduces additional congestion. This occurs through interactions with the distal terminus of the diene, as well as with the approaching dienophile, thereby raising the activation barrier. As shown in Figure 5b, this effect is largely independent of the identity of the substituent R at C^2^.

**2 usch2:**
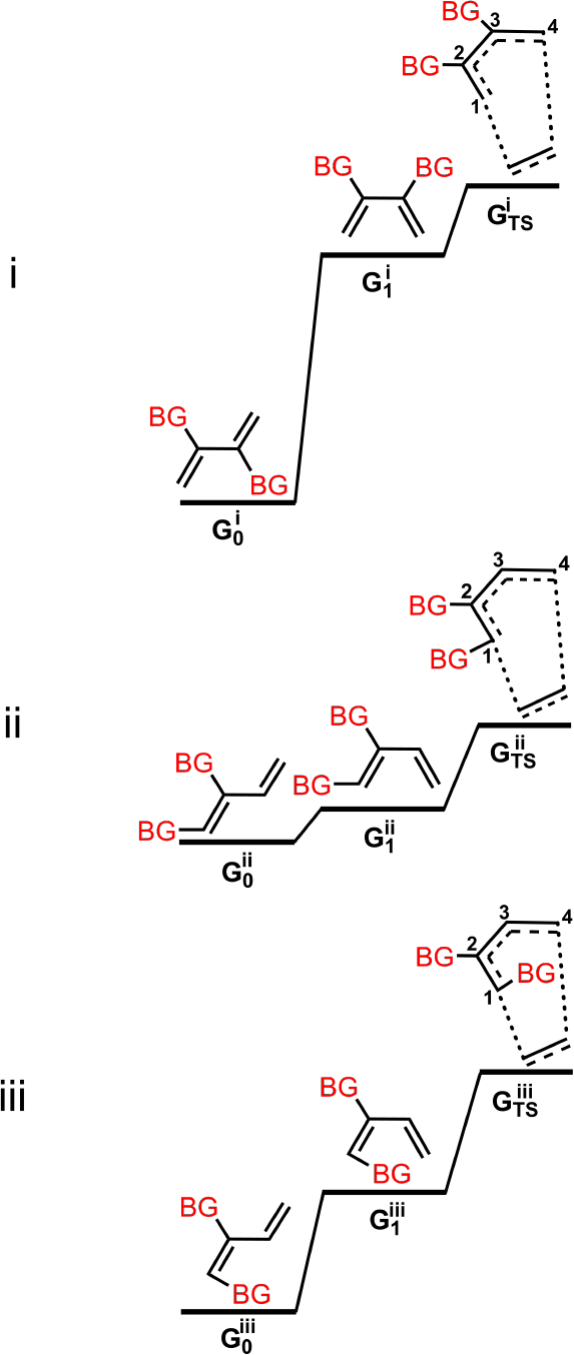
Configurations with Bulky Substituents at Carbon Positions 1 and 2 or 2 and 3 of the Diene

### Effect of EDG and EWG across All Reactive Sites, Especially at C^5^ and C^6^

As illustrated in Figure 3, the min_LUMO–HOMO_ parameter ranks among the top electronic features influencing the activation barrier in the machine learning model. As previously noted, the HOMO–LUMO gap has long been recognized as a central determinant of Diels–Alder reactivity, with its significance reported consistently since 1973.^10–13,27,28^ The most favorable arrangement for minimizing the HOMO–LUMO gap occurs when an EWG is positioned on one component and an EDG on the other. To investigate this effect, three representative substituents were selected: CN (strong EWG, σ_*p*_ = 0.66), H (neutral, σ_*p*_ = 0.00), and OMe (EDG, σ_*p*_ = −0.27).

As shown in Figure 6, a decrease in the energy gap between the HOMO of the diene and the LUMO of the dienophile or between the HOMO of the dienophile and the LUMO of the diene is associated with a lower activation barrier in the Diels–Alder reaction. This trend is further supported by SHAP analysis, which highlights the strong influence of frontier orbital interactions. The only notable deviations from this pattern are observed in cases where the diene carries hydrogen as the sole substituent. As previously discussed, such systems do not experience significant steric congestion in the transition state. For example, in the case of R = H (Figure 6a), the min_LUMO–HOMO_ gap is larger than that of R = OMe (6.14 and 5.02 eV, respectively), yet the calculated activation barrier for R = H is slightly lower.

**Figure 6:**
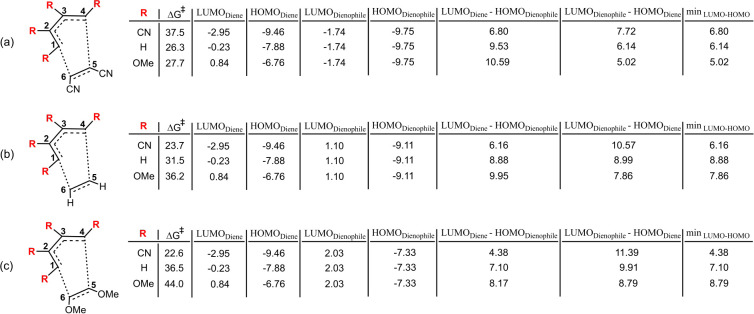
Influence of CN, H, and OMe substitutions at C^5^ and C^6^, and variations at positions C^1^–C^4^ on activation energies and HOMO–LUMO gap values: including LUMO_diene_–HOMO_dienophile_ and LUMO_dienophile_–HOMO_diene_ interactions. The minimum value between the LUMO–HOMO gaps (LUMO_diene_–HOMO_dienophile_ and LUMO_dienophile_–HOMO_diene_ is reported in the final column for each substitution pattern. All $\Delta G^{‡}$ values are given in kcal/mol.

While electronic parameters are widely acknowledged as key factors influencing the activation barriers of Diels–Alder reactions, the preceding discussion makes it clear that the presence of an electron-donating or electron-withdrawing group on either the diene or the dienophile, on its own, does not determine the direction of the change in the activation barrier (see Figures S4 and S5). Instead, the outcome is determined by the relative energies and alignment of the frontier molecular orbitals of both components. This observation is supported by the SHAP analysis, which identifies min_LUMO–HOMO_, along with steric descriptors, such as substituent volume, as the most impactful features. A minor contribution from the electronic character of the substituents, as quantified by the Hammett σ_*p*_ parameter, is also observed. For example, more electron-donating groups are associated with slightly higher activation barriers. This effect, although significantly less pronounced than those caused by steric bulk or min_LUMO–HOMO_, may stem from substituents like *tert*-butyl. This group exhibits both electron-donating character (σ_*p*_ < 0) and considerable steric demand, and the elevated activation barriers observed in *tert*-butyl-substituted systems may arise from steric congestion rather than from its electron-donating nature.

## Conclusion

This study integrates DFT calculations with interpretable machine learning models to investigate the steric and electronic features that influence activation barriers in uncatalyzed Diels–Alder reactions. While the minimum of the two frontier energy gaps (LUMO_diene_–HOMO_dienophile_ and LUMO_dienophile_–HOMO_diene_) has long served as a key electronic descriptor in orbital-based reactivity models, our results demonstrate that this parameter alone is insufficient to account for reactivity trends across diverse substitution patterns.

Specifically, we show that bulky substituents at the internal diene positions (C^2^ and C^3^) can dramatically increase the activation barrier by enforcing high-energy conformations characterized by significant steric congestion. This effect is spatially localized and highly dependent on the relative orientation of substituents, highlighting the importance of conformational dynamics in DA transition state geometries. SHAP-based feature attribution confirms the dominant influence of the steric volume at these positions, ranking it above classical electronic descriptors in predicting activation free energies.

By evaluating electronic and steric effects across 1000 representative Diels–Alder structures, our ML framework extends beyond conventional frontier molecular orbital theory, delivering predictive insight into nuanced structure–reactivity relationships and establishing a foundation for rational substituent design aimed at improving selectivity and reaction rates.

## Computational Details

The initial molecular structures were systematically generated using Maestro (Schrödinger, LLC). For any species in which the presence of different conformers was likely based on the nature of the substituents, a manual conformational search was performed to identify the most stable conformer used in the calculations. Gaussian jobs were automatically submitted, and their outputs were processed through in-house Python scripts. We employed the random forest regression (RFR) and gradient boosting (GB) machine learning model, implemented using the Scikit-learn package^36^ in Python.

All structures were fully optimized using Gaussian 16 ^37^ at the M06-2X level of theory.^38^ The reliability of the chosen DFT methodology is supported by recent large-scale benchmark studies on Diels–Alder reactions, which identify M06-2X-based approaches as among the most accurate single functionals for predicting activation free energies.^39^ Solvent effects were incorporated via the SMD solvation model^40^ employing dichloromethane as the solvent throughout. Geometry optimizations and subsequent frequency calculations were carried out using the 6-31G­(d) basis set.^41^ Transition states were located by using the Berny optimization algorithm. To refine the electronic energies obtained at the SMD/M06-2X/6-31G­(d) level, single-point energy calculations were performed using the def2-TZVP basis set.^42^

A tight convergence criterion and an ultrafine integration grid were employed to enhance the accuracy of all of the calculations. The solution-phase Gibbs free energy for each species was computed according to the following expression:

1 $$G=E\left(BS2\right)+G\left(BS1\right)-E\left(BS1\right)+\Delta G^{1atm\to 1M}$$

where Δ*G* ^1atm→1M^ = 1.89 kcal/mol is the free-energy change for compression of 1 mol of an ideal gas from 1 atm to the 1 M solution-phase standard state. For a representative subset of reactions, BSSE corrections were applied at the M06-2X/def2-TZVP level, and benchmark single-point energy calculations were also performed at the DLPNO–CCSD­(T)/CBS­(2/3) level of theory, with extrapolated correlation energies using the extrapolate­(2/3,def2) options as implemented in ORCA version 6.0.0, to ensure the reliability of the computed activation barriers (for details, see the Supporting Information).

## Supplementary Material



## Data Availability

The data underlying this study are openly available in GitHub at https://github.com/mahdianamir/DielsAlder-ML-Data.

## Notes

Additional computational results (PDF)
