# Supplementary material for: A Machine Learning-Guided Study of Structure–Reactivity Relationships in Diels–Alder Cycloadditions
Source: J Org Chem. 2026 Jan 7;91(3):1329–36. doi: 10.1021/acs.joc.5c02349 (PMC12836318; doi:10.1021/acs.joc.5c02349)
Supplement: Supplementary file 1 [file jo5c02349_si_001.pdf]

A Machine Learning Guided Study of Structure-Reactivity Relationships in Diels–Alder Cycloadditions

Amir Mahdian[a], Kaveh Farshadfar*[a], Kari Laasonen*[a]

[a] *Department of chemistry and material science, School of chemical engineering, Aalto University, 02150 Espoo, Finland*

Contents

0.1 Summary of Quantum Chemical Descriptors for Predictive Modeling S2

0.2 Evaluation and Hyperparameter Optimization of Machine Learning Models for Predicting $\Delta G^{\ddagger}_{\text{min}}$ S4

0.3 Analysis of Random Forest model Predictions and SHAP Interpretation for Normal and Inverse Diels–Alder Reactions . S7

0.4 Electronic Influence of CN, F, and OMe Substitution at C^1, C^3, and C^4 on Diels–Alder Activation Barriers: Controlling Reactivity through EWG and EDG S10

0.5 Electronic Effects of CN, F, and OMe Substitution at C^2, C^3, and C^4 on activation energy: Influence of EWG and EDG on Diels–Alder Reactivity S12

0.6 Verification of Descriptor Stability Using SHAP Analysis on Training and Test Sets S14

Email addresses: `kaveh.farshadfar@aalto.fi` (Kaveh Farshadfar*), `kari.laasonen@aalto.fi` (Kari Laasonen*)

0.1. Summary of Quantum Chemical Descriptors for Predictive Modeling

The corresponding 37 electronic and steric descriptors used for model training are listed and summarized in Table S1. These descriptors include frontier molecular orbital energies (HOMO and LUMO) for both the diene and the dienophile, along with various energy gaps between them, which are essential for evaluating electronic compatibility. Orbital interactions are further described by analyzing the p_z orbital occupancies of the six carbon atoms involved in the cycloaddition. Electronic effects are quantified using Hammett σ_p parameters and natural population analysis (NPA) charges, providing insights into the electron-donating or -withdrawing characteristics of each substituent. Steric effects were incorporated through substituent volume (estimated as the solvent-accessible space excluding regions occupied by atomic structure) and through sterimol parameters, which quantify the size and shape of substituents based on their conformational ensemble and spatial profile[1] , thus capturing both steric demand and conformational diversity .

Table S1: Electronic and Steric Descriptors Employed in Machine Learning Models for Predicting Diels–Alder Reactivity

**Name of descriptor**	**Explanation**
p_z Orbitals on Carbons 1–6	The electron density associated with the p_z orbitals on carbons 1–6
σ_p on Carbons 1-6	A quantitative descriptor of the electron-withdrawing or electron-donating character of substituents attached to carbons 1–6
NPA charges on carbons 1–6	Atomic charges on carbons 1–6 calculated using Natural Population Analysis (NPA)
Sterimol Parameters for Substituents on Carbons 1–6	A set of multidimensional steric descriptors used to quantify the size and shape of substituents on carbons 1–6
Substituent Volumes on Carbons 1–6	Solvent-accessible volume of the substituent on carbons 1–6, excluding regions blocked by the atomic framework
The LUMO energy of the diene	Energy of the diene's lowest unoccupied molecular orbital

**Name of descriptor**	**Explanation**
The HOMO energy of the Diene	Energy of the diene's highest occupied molecular orbital
The LUMO energy of the Dienophile	Energy of the dienophile's lowest unoccupied molecular orbital
The HOMO energy of the Dienophile	Energy of the dienophile's highest occupied molecular orbital
The $LUMO_{dien}$–$HOMO_{dienophile}$ energy	The energy gap between the Lowest Unoccupied Molecular Orbital (LUMO) of the diene and the Highest Occupied Molecular Orbital (HOMO) of the dienophile.
The $LUMO_{dienophile}$–$HOMO_{dien}$ energy	The energy gap between the Lowest Unoccupied Molecular Orbital (LUMO) of the dienophile and the Highest Occupied Molecular Orbital (HOMO) of the diene
The $\min_{LUMO-HOMO}$ energy	The minimum energy difference between the Lowest Unoccupied Molecular Orbital (LUMO) and the Highest Occupied Molecular Orbital (HOMO) of the reacting species

0.2. Evaluation and Hyperparameter Optimization of Machine Learning Models for Predicting $\Delta G^{\ddagger}_{min}$

In this study, a range of machine learning algorithms—including linear regression, decision trees, support vector machines (SVM), k-nearest neighbors (KNN), gradient boosting, and random forests—was evaluated for predicting $\Delta G^{\ddagger}_{\text{min}}$ using a descriptor set of 37 electronic and steric molecular features. The predictive performance of each model is summarized below to enable direct comparison of their applicability to modeling reactivity in Diels–Alder cycloadditions.

Linear Regression: MAE: 3.48, R^2: 0.72
Decision Tree: MAE: 3.58, R^2: 0.68
Support Vector Machine: MAE: 3.16, R^2: 0.66
K-Nearest Neighbors: MAE: 3.05, R^2: 0.76
Gradient Boosting: MAE: 2.06, R^2: 0.89
Random Forest: MAE: 2.47, R^2: 0.845

Among the evaluated algorithms, random forest and gradient boosting regression demonstrated the highest predictive performance, as reflected by their elevated R^2 values and low mean absolute errors (MAEs). Accordingly, these two models were selected for further analysis and interpretation in the main text.

Hyperparameter Optimization Procedure: Hyperparameters of the Random Forest and Gradient Boosting regression models were optimized using a grid search combined with 10-fold cross-validation. The parameter ranges were selected based on prior literature and preliminary tuning experiments. The final optimized values are summarized in Tables S2 and S3.

Table 2. Architecture of the Random Forest Regression Model.

**Parameter**	**Value / Setting**
Number of Estimators (n_estimators)	600
Bootstrap	True
CCP Alpha	0.0
Criterion	squared_error
Maximum Depth	None
Maximum Features	sqrt
Maximum Leaf Nodes	None
Maximum Samples	None
Minimum Impurity Decrease	0.0
Minimum Samples at a Leaf Node	1
Minimum Samples to Split an Internal Node	2
Minimum Weight Fraction Leaf	0.0
Monotonic Constraints	None
Out-of-Bag (OOB) Estimation	True
Random State	42
Verbose	0
Warm Start	False
Cross-Validation	10-fold cross-validation for hyperparameter tuning

Table S3. Architecture of the Gradient Boosting Regression Model.

**Parameter**	**Value / Setting**
Number of Estimators (n_estimators)	100
Learning Rate	0.1
Maximum Depth	3
Random State	Default
Cross-Validation	10-fold cross-validation for hyperparameter tuning

Descriptor Correlation Analysis: Pairwise Pearson correlation coefficients were calculated for all thirteen DFT derived molecular descriptors in order to evaluate potential redundancy among the features employed in the SHAP analysis. The complete correlation matrix is reported in Table

S4. Overall, the descriptors exhibit relatively low mutual correlation, with the highest absolute coefficient being $|r| = 0.50$. As correlation values above $|r| = 0.8$ are commonly taken to indicate significant redundancy, no meaningful multicollinearity was identified, and all descriptors were retained for subsequent SHAP based interpretation.

Table S4. Pearson correlation matrix of all molecular descriptors used in SHAP analysis.

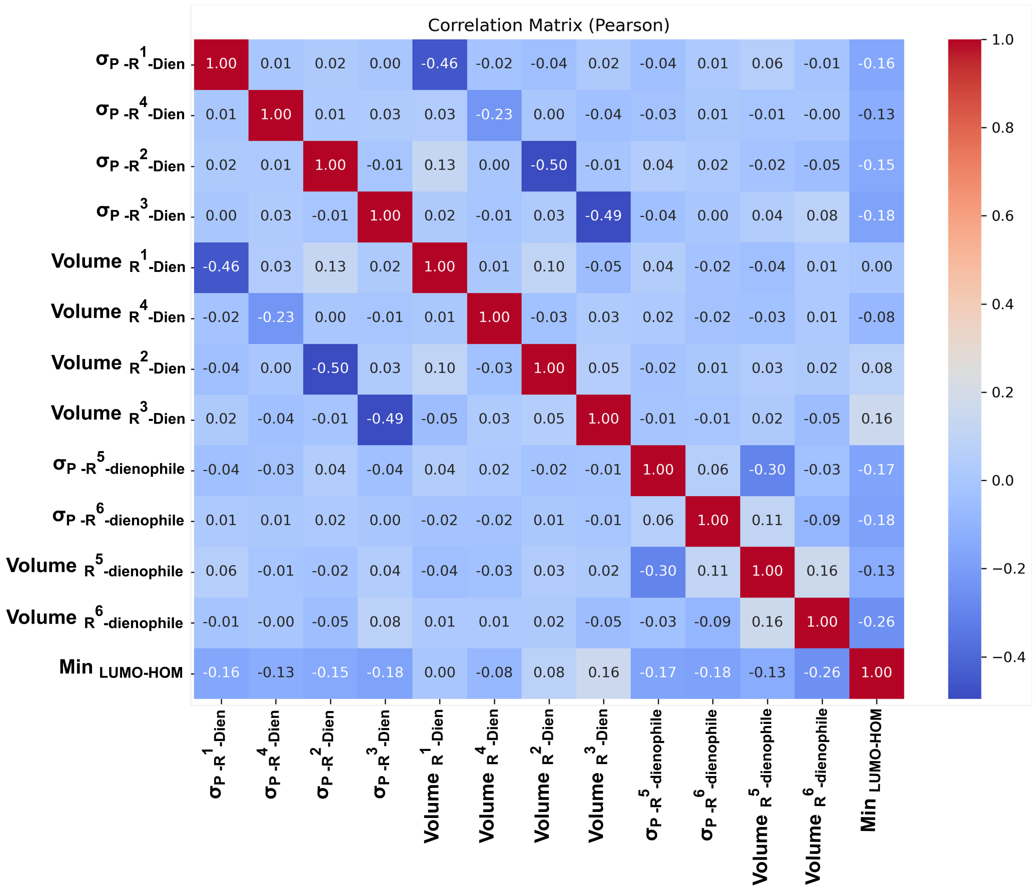

0.3. Analysis of Random Forest model Predictions and SHAP Interpretation for Normal and Inverse Diels–Alder Reactions

As illustrated in Figure S1, approximately 500 molecules were classified as undergoing normal Diels–Alder reactions and were accurately predicted by the RF model. As noted in the main text, any molecule exhibiting electron transfer from the diene to the dienophile fall into this category and can be accurately predicted using the trained model. Consistent with these findings, SHAP analysis (Figure S2) highlights the dominant influence of the volumes at C^2 and C^3 positions in determining reactivity. The next most significant features were the Hammett σ_p parameters of the substituents at C^5 and C^6-positions on the dienophile. Notably, strongly EWG such as CN, ($\sigma_p = 0.66$) significantly reduce the LUMO–HOMO energy gap, thereby lowering the activation barrier. This observation supports the well-established correlation between the $LUMO_{dienophile}$–$HOMO_{diene}$ energy gap and the activation energy, wherein a narrower gap corresponds to a lower reaction barrier.

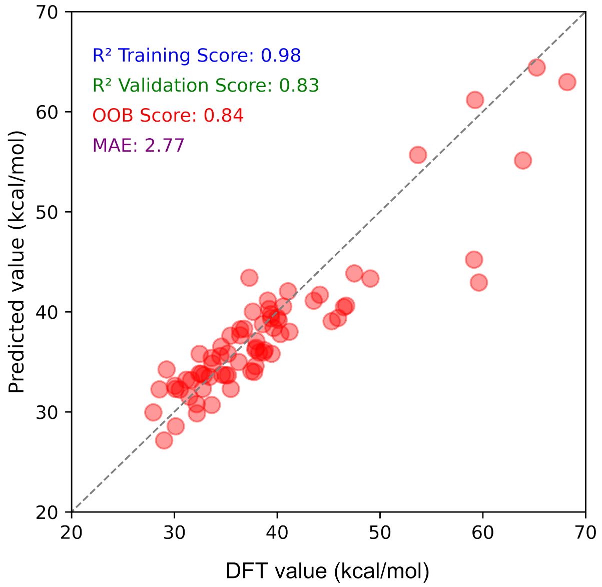

Figure S1: Scatter plots comparing the DFT-calculated $\Delta G^{\ddagger}_{min}$ values with the predicted values from the trained random forest model for the Normal Diels–Alder reaction, where attribute features are used as the basis for predictions within the training dataset.

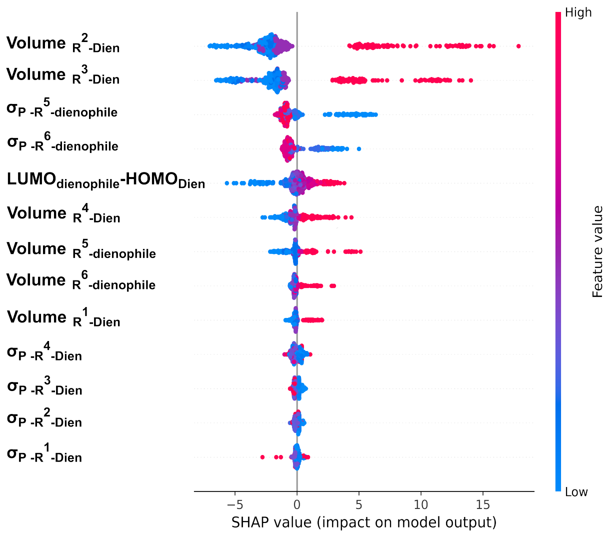

Figure S2: SHAP value plot for the Normal Diels–Alder reaction, depicting the contribution of individual features across different samples. The color gradient represents feature values, with blue indicating lower values and red corresponding to higher values.

Approximately half of the remaining molecules were identified as undergoing inverse Diels–Alder reactions. As shown in Figure S3, the RF model demonstrated high predictive accuracy for this reaction class as well. These results highlight the utility of the model as a practical tool for predicting reaction kinetics across mechanistically diverse Diels–Alder pathways. In this case, SHAP analysis (Figure S4) again identified the steric volumes of substituents at C^2 and C^3 as the most influential features. However, in contrast to the normal Diels–Alder pathway, the volumes of substituents at C^5 and C^6 on the dienophile were also found to be highly significant.

As in the case of normal reactions, substituents that reduce the energy gap between the $LUMO_{diene}$ and $HOMO_{dienophile}$ tend to accelerate the reaction by enhancing frontier orbital interactions. In contrast, the σ_p parameters of the substituents had a diminished overall effect in this model. Nonetheless, substituents with high σ_p values, indicative of strong electron-withdrawing character, still contributed to narrowing the frontier orbital gap and consequently lowering the activation barrier.

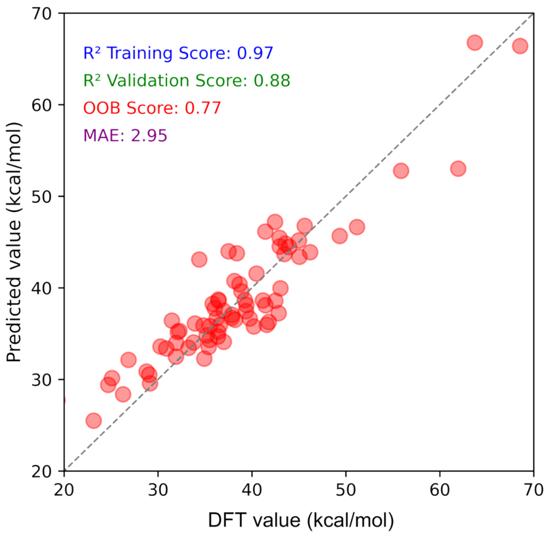

Figure S3: Scatter plots illustrating the relationship between the DFT-calculated $\Delta G^{\ddagger}_{min}$ values and the predicted values from the trained random forest model for the Inverse Diels–Alder reaction, using attribute features as the basis for predictions within the training dataset.

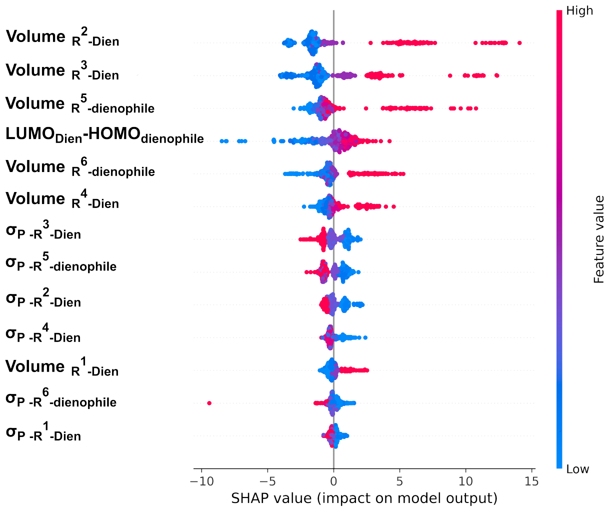

Figure S4: SHAP value plot for the Inverse Diels-Alder reaction, highlighting the contribution of individual features across various samples. The color gradient represents feature magnitudes, with blue denoting lower values and red corresponding to higher values

0.4. *Electronic Influence of CN, F, and OMe Substitution at C^1, C^3, and C^4 on Diels–Alder Activation Barriers: Controlling Reactivity through EWG and EDG*

To gain mechanistic insight into the Diels–Alder reaction, a set of substituents was introduced, and their effects on reactivity were quantitatively assessed. The results, summarized in Figures S5, S6, and S7, reveal consistent electronic trends across all three molecular systems. As noted in previous computational studies,[2] incorporation of a CN group on the dienophile substantially decreases the activation barrier. CN reduces electron density of the dienophile, stabilizes its LUMO, and facilitates interaction with the diene HOMO, thereby decreasing the activation energy of the cycloaddition. As illustrated in the figures 6, the presence of additional EWGs consistently reduces the activation barrier across all substitution patterns, accelerating the reaction. Conversely, EDG substitution raises the barrier, likely by diminishing the electrophilic character of the dienophile or diene, resulting in slower reaction kinetics.

To assess the reliability of the M06-2X/def2-TZVP level of theory used in this study, benchmark single-point energy calculations were carried out at the DLPNO-CCSD(T)/CBS(2/3) level for a representative subset of Diels–Alder transition states. The resulting activation barriers are in very good agreement with the M06-2X values, with mean deviations of less than 2 $\text{kcal} \cdot \text{mol}^{1}$. BSSE corrections were also evaluated for selected systems, confirming that basis-set superposition errors at the M06-2X/def2-TZVP level are small and do not affect the observed trends in reactivity.

As inferred from the SHAP analysis in Figure 3, incorporation of additional EWGs within the molecular framework generally lowers the activation barrier, stabilizes the transition state, and promotes product formation. In contrast, EDGs tend to raise the activation energy, destabilize the transition state, and reduce overall reactivity.

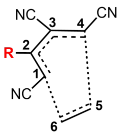

R	$\min_{\text{LUMO-HOMO}}$	$\Delta G^{\ddagger}$	$\Delta G^{\ddagger}_{\text{CCSD}}$	BSSE
CN	6.15	23.71	24.76	2.3
H	6.69	27.06	27.56	2.4
F	6.40	27.46	28.37	2.4
OMe	6.70	28.39	29.20	2.5
^{t}Bu	7.03	33.06	33.77	2.5

Figure S5: Effect of CN substitution at carbons 1, 3, and 4, with varying substituents at carbon 2, on activation energy. The minimum value between the LUMO–HOMO gaps ($\text{LUMO}_{\text{diene}}$ – $\text{HOMO}_{\text{dienophile}}$ and $\text{LUMO}_{\text{dienophile}}$ – $\text{HOMO}_{\text{diene}}$) is reported for each substitution pattern. R^5 and R^6 are set to H. All $\Delta G^{\ddagger}$ values are given in kcal/mol and were computed at the def2-TZVP and CCSD(T) level with BSSE correction applied.

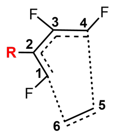

R	$\min_{\text{LUMO-HOMO}}$	$\Delta G^{\ddagger}$	$\Delta G^{\ddagger}_{\text{CCSD}}$	BSSE
CN	8.27	29.11	31.26	3.0
H	9.27	31.59	33.15	3.2
F	9.23	33.18	35.17	3.2
OMe	9.12	34.02	35.16	3.2
^{t}Bu	9.24	36.21	37.25	3.3

Figure S6: Effect of F substitution at carbons 1, 3, and 4, with varying substituents at carbon 2, on activation energy. The minimum value between the LUMO–HOMO gaps ($\text{LUMO}_{\text{diene}}$ – $\text{HOMO}_{\text{dienophile}}$ and $\text{LUMO}_{\text{dienophile}}$ – $\text{HOMO}_{\text{diene}}$) is reported for each substitution pattern. R^5 and R^6 are set to H. All $\Delta G^{\ddagger}$ values are given in kcal/mol and were computed at the def2-TZVP and CCSD(T) level with BSSE correction applied.

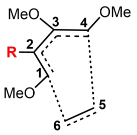

R	$\min_{\text{LUMO-HOMO}}$	$\Delta G^{\ddagger}$	$\Delta G^{\ddagger}_{\text{CCSD}}$	BSSE
CN	8.23	32.87	33.24	3.2
H	7.88	33.86	33.63	3.3
F	8.01	35.92	35.83	3.3
OMe	7.75	36.41	36.37	3.2
^{t}Bu	7.89	40.25	39.26	3.4

Figure S7: Effect of OMe substitution at carbons 1, 3, and 4, with varying substituents at carbon 2, on activation energy. The minimum value between the LUMO–HOMO gaps ($\text{LUMO}_{\text{diene}}$ – $\text{HOMO}_{\text{dienophile}}$ and $\text{LUMO}_{\text{dienophile}}$ – $\text{HOMO}_{\text{diene}}$) is reported for each substitution pattern. R^5 and R^6 are set to H. All $\Delta G^{\ddagger}$ values are given in kcal/mol and were computed at the def2-TZVP and CCSD(T) level with BSSE correction applied.

0.5. *Electronic Effects of CN, F, and OMe Substitution at C^2, C^3, and C^4 on activation energy: Influence of EWG and EDG on Diels–Alder Reactivity*

The influence of substituents attached to carbon 1 is less pronounced compared to those at carbon 2, as discussed in the main text. However, it remains evident that electron-withdrawing groups such as CN or F can significantly lower the activation barrier by reducing the HOMO–LUMO energy gap. As shown in Figure S8, the barrier energy in the presence of a CN substituent ranges from approximately 23 to 28 kcal/mol. When the electron-withdrawing strength is reduced—for example, by replacing CN with F—the energy gap increases, resulting in a higher activation barrier in the range of 32 to 35 kcal/mol (Figure S9). Furthermore, introducing an electron-donating group such as OMe at the same position leads to a substantial widening of the HOMO–LUMO gap, elevating the barrier energy to 35–39 kcal/mol, as demonstrated in Figure S10. These computed trends are further supported by the DLPNO-CCSD(T)/CBS(2/3) benchmark calculations and BSSE-corrected energies reported in Sections S4–S5, confirming the reliability of the activation barriers discussed here.

As illustrated in Figures S8 and S9, a consistent trend was observed for electron-withdrawing substituents: their presence led to a marked reduction in activation energy. This pattern was evident across systems containing CN and F groups, reflecting the ability of electron-withdrawing substituents to stabilize the transition state. In contrast, electron-donating groups such as OMe resulted in significantly higher activation barriers, indicating a general increase in reactivity resistance under these electronic conditions. Among all substituents examined, the highest activation barrier was observed for the ^{t}Bu group, which is attributed primarily to steric hindrance rather than electronic effects. However, the observed trend deviates in the case of electron-donating substituents, as illustrated in Figure S10. When an electron-donating group such as OMe is positioned at carbon 1, it activates the diene by increasing its electron density, thereby enhancing its ability to interact with the dienophile. This electronic effect is consistent with the behavior observed in normal Diels–Alder reactions, where donor-substituted dienes typically exhibit lower activation barriers. Nevertheless, the overall trend for other substituents, as shown in Figures S8 and S9, does not follow this expected pattern. In fact, many electron-donating groups are associated with relatively high activation barriers in this system.

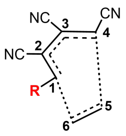

R	$\min_{\text{LUMO-HOMO}}$	$\Delta G^{\ddagger}$	$\Delta G^{\ddagger}_{\text{CCSD}}$	BSSE
CN	6.15	23.71	24.76	2.3
CHO	6.21	24.24	25.03	2.6
H	6.79	24.81	26.78	2.3
COOMe	6.41	25.48	25.14	2.7
F	6.86	25.62	27.44	2.7
Me	6.93	26.74	27.69	2.4
Ph	6.68	27.51	27.95	2.6
OMe	7.08	28.41	28.88	2.8
^{t}Bu	6.99	28.61	28.40	2.6

Figure S8: Influence of CN substitution at carbons 2, 3, and 4, with varying substituents at carbon 1, on activation energy. The minimum value between the LUMO–HOMO gaps ($\text{LUMO}_{\text{diene}} - \text{HOMO}_{\text{dienophile}}$ and $\text{LUMO}_{\text{dienophile}} - \text{HOMO}_{\text{diene}}$) is reported for each substitution pattern. R^5, R^6 = H. All $\Delta G^{\ddagger}$ values are given in kcal/mol and were computed at the def2-TZVP and CCSD(T) level with BSSE correction applied.

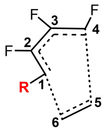

R	$\min_{\text{LUMO-HOMO}}$	$\Delta G^{\ddagger}$	$\Delta G^{\ddagger}_{\text{CCSD}}$	BSSE
CN	7.85	32.12	33.05	2.9
CHO	7.73	32.44	33.14	3.2
H	9.06	33.13	34.80	2.9
F	8.98	33.18	35.17	3.2
COOMe	8.06	33.50	33.78	3.3
Me	9.31	34.11	35.04	3.0
Ph	8.33	34.23	34.68	3.2
OMe	9.48	34.93	36.17	3.3
^{t}Bu	9.32	35.23	35.04	3.2

Figure S9: Influence of F substitution at carbons 2, 3, and 4, with varying substituents at carbon 1, on activation energy. The minimum value between the LUMO–HOMO gaps ($\text{LUMO}_{\text{diene}} - \text{HOMO}_{\text{dienophile}}$ and $\text{LUMO}_{\text{dienophile}} - \text{HOMO}_{\text{diene}}$) is reported for each substitution pattern. R^5, R^6 = H. All $\Delta G^{\ddagger}$ values are given in kcal/mol and were computed at the def2-TZVP and CCSD(T) level with BSSE correction applied.

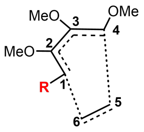

R	$\min_{\text{LUMO-HOMO}}$	$\Delta G^{\ddagger}$	$\Delta G^{\ddagger}_{\text{CCSD}}$	BSSE
OMe	7.87	35.93	34.93	3.2
F	8.18	36.09	36.57	3.3
CN	8.42	37.12	36.41	3.0
H	8.16	37.47	36.85	3.3
CHO	8.35	37.60	36.12	3.3
Me	7.99	37.76	37.66	3.0
Ph	7.78	38.28	37.02	3.2
COOMe	8.32	38.46	37.02	3.4
^{t}Bu	8.02	39.21	38.64	3.3

Figure S10: Influence of OMe substitution at carbons 2, 3, and 4, with varying substituents at carbon 1, on activation energy. The minimum value between the LUMO–HOMO gaps ($\text{LUMO}_{\text{diene}} - \text{HOMO}_{\text{dienophile}}$ and $\text{LUMO}_{\text{dienophile}} - \text{HOMO}_{\text{diene}}$) is reported for each substitution pattern. R^5, R^6 = H. All $\Delta G^{\ddagger}$ values are given in kcal/mol and were computed at the def2-TZVP and CCSD(T) level with BSSE correction applied.

0.6. *Verification of Descriptor Stability Using SHAP Analysis on Training and Test Sets*

To evaluate the stability of the descriptor importance rankings, SHAP analyses were performed on the full dataset, the training subset, and the independent test subset. In all three analyses, the same three descriptors consistently appeared as the most important features. This result demonstrates that the dominant descriptors identified for the final model are robust with respect to data splitting and are intrinsic to the underlying reaction chemistry. Representative SHAP summary plots for the training and test sets are provided below.

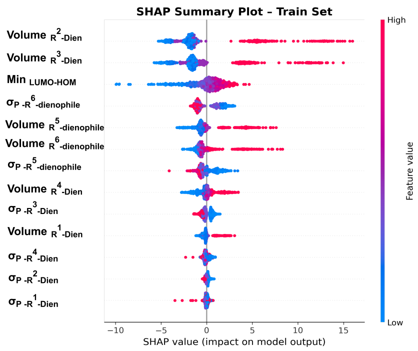

Figure S11: SHAP values (kcal/mol) representing feature contributions for the training subset used in model fitting. Color gradients indicate feature magnitudes (blue = low, red = high).

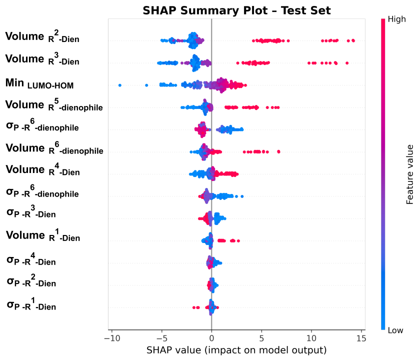

Figure S12: SHAP values (kcal/mol) illustrating feature contributions for samples in the independent test set. Color gradients reflect feature-value magnitudes (blue = low, red = high).
